# Partial Pulpotomy as an Applicable Treatment Option for Cariously Exposed Posterior Permanent Teeth: A Systematic Review of Randomized Clinical Trials

**DOI:** 10.7759/cureus.26573

**Published:** 2022-07-05

**Authors:** Sarah S Albaiti, Raghad F Albishri, Moeid T Alhowig, Wessam I Tayyar, Nouf F Alqurashi, Faisal T Alghamdi

**Affiliations:** 1 General Dentistry, Faculty of Dentistry, King Abdulaziz University, Jeddah, SAU; 2 Oral Biology, Faculty of Dentistry, King Abdulaziz University, Jeddah, SAU

**Keywords:** vital pulp, pulpotomy, pulpitis, randomized clinical trial, dental pulp exposure, dental caries

## Abstract

The major focus of this systematic review is to assess how effective partial pulpotomy is in managing carious vital pulp exposures in permanent posterior teeth. An electronic search for studies published between January 2011 and December 2021 was conducted using the following databases: PubMed, Scopus, Google Scholar, and Web of Science. The Preferred Reporting Items for Systematic Reviews and Meta-Analyses (PRISMA) criteria were followed during the search process. We selected human randomized clinical trials (RCTs) that investigated the success rate of partial pulpotomy for the treatment of cariously exposed vital permanent posterior teeth and the success rate of decayed exposed vital human permanent posterior dentition managed with a partial pulpotomy. Exclusively, randomized clinical trial papers were considered for assessment. The Cochrane Collaboration’s tool was applied to assess the risk of bias. Four papers were selected for the final analysis from the 321 identified during the initial search. Our results showed that after six, 12, and 24 months of follow-up, the success rate was 94%, 93%, and 90%, respectively. The preoperative pulp state was the only significant predictive factor. Teeth with the presumptive diagnosis of irreversible pulpitis had the worse outcome. The treatment outcome was not influenced by the final restoration, pulp capping agent, apex closure, or patient age. Finally, the available data indicated that partial pulpotomy showed a high success rate in treating cariously exposed permanent posterior teeth for up to 24 months. When assessing the effectiveness of a partial pulpotomy, six months of maintenance is deemed adequate. To enhance treatment success, additional clinical and radiological measures are needed.

## Introduction and background

The International Caries Consensus Collaboration conference in Leuven, Belgium, in 2015 presented suggestions that preserving pulp vitality must be a top priority in the management of severe carious lesions. Whenever feasible, pulpal exposures should be avoided using minimally invasive procedures, such as partial or selective caries removal [1]. Even with a more cautious and conservative approach, pulp exposure is sometimes unavoidable [2]. In such instances, endodontic treatment (ET) is regarded as the preferred treatment, due to its significant success and long-term survival rate [3,4].

For the treatment of pulp exposures in vital teeth, a more cautious and conservative approach should be explored. As an alternative to ET, vital pulp therapy treatment methods have been tried. Options include indirect and direct pulp capping, and partial and complete pulpotomy [5,6]. By preserving the pulpal tissues, these procedures aim to facilitate the functional and structural recovery of the pulp-dentin complex and thereby extend the longevity of the tooth [7,8]. Furthermore, these treatment options are often more cost-effective and much less technique-sensitive than root canal treatment (RCT) [9].

Treatment for each patient is typically decided following the preliminary diagnosis. Uncertainty remains concerning the factors that should be considered when selecting the preferred therapy. According to one study, the efficiency of tissue renewal or healing is controlled by the degree of inflammatory irritations in the pulpal tissues [10]. In profound carious lesions, the inflammatory irritations on the surface layers of pulpal tissues are often more prominent than those in the deeper layers. Regardless of blood vessel dilatation, the pulp tissues in the root canal are usually normal [10,11].

Partial pulpotomy involves removing 2-3 mm of pulpal tissues underneath the exposure site. This treatment option is indicated when the pulp tissues are exposed due to injury or impaction [12]. Compared to sealing the injured pulpal tissues via direct pulp capping, the surgical amputation of the affected pulpal tissues before sealing the exposure may increase the likelihood of recovery and healing [13,14]. Partial pulpotomy may be preferable to complete pulpotomy in terms of recovery due to the preservation of cell-rich tissues in the coronal pulp region. Complete pulpotomy involves removing the entire coronal pulp, which increases the likelihood of canal obliteration [10,15].

According to available research, the effectiveness of vital pulp therapies differs greatly between studies [15,16]. To accurately select an acceptable treatment approach for cariously exposed teeth, substantial evidence is critical. Unfortunately, the literature shows a dearth of high-level evidence, with only one systematic review, conducted nearly a decade ago [14].

A recent study [17] observed that there were various drawbacks of the coronal pulpotomy as a treatment option for cariously exposed permanent posterior teeth with closed apices, including the pooling of follow-up intervals, conflicting conditions of success, and diverse carious lesion removal procedures. For example, stepwise excavation of caries followed by sealing the tooth with mineral trioxide aggregate (MTA) was considered the treatment of choice for cariously exposed teeth in only two studies. As an alternative to calcium hydroxide (CH), cements such as hydraulic calcium silicate are frequently recommended for vital pulp therapy since they promptly promote the formation of a minimally porous hard tissue layer [18,19].

Over the previous 10 years, there is a considerable increase in the number of clinical trials on this topic. Some studies have investigated the use of MTA or MTA-like cements as the pulp capping agent [16,20-23], thus contributing more evidence for assessing the efficacy of this treatment strategy. This systematic review aimed to assess the success rate of partial pulpotomy for the treatment of permanent posterior teeth with carious vital pulp exposures by evaluating the available randomized clinical trial (RCT) studies because these teeth are more prone to caries problems compared to other teeth and also few studies focused on this type of tooth.

## Review

Material and methods

This systematic review was conducted according to the Preferred Reporting Items for Systematic Reviews and Meta-Analyses (PRISMA) criteria [24].

Review Question

The review question is as follows: “Is partial pulpotomy an applicable treatment option for cariously exposed posterior permanent teeth?”

Search Strategy

Five reviewers did their own electronic searches in the following databases: PubMed, Scopus, Web of Science, and Google Scholar. The following key terms were used: dental pulp, miniature pulpotomy, partial pulpotomy, and pulp curettage. Publications in the English language published between January 1, 2011, and December 31, 2021, were searched. The reference lists of the selected studies were reviewed to identify other relevant studies. When there was a question concerning a study, the authors were contacted for clarification by e-mail.

Inclusion and Exclusion Criteria

This current study was designed to identify clinical papers that assessed the success rate of partial pulpotomy in the management of carious vital pulp exposure in permanent posterior teeth. Exclusively, published randomized clinical trials from the previous 10 years (2011-2021) were identified and analyzed. To be included, a study must include a sample size of at least 10 teeth, a minimum follow-up of six months, and a minimum follow-up rate of 80%.

The success rate must be accessible or quantifiable based on the presented data. For example, some studies have used pain, percussion, and palpation tenderness as indicators of treatment outcomes. Clinical and radiographical evidence of inflammatory irritation, necrosis, or root resorption has also been considered. In this review, the success rate was evaluated based on the clinical and radiographical outcomes after the partial pulpotomy treatment at different follow-ups (six months, one year, and two years).

The exclusion criteria included reviews, case reports, observational studies, and in vitro/in vivo studies; studies with unclear follow-up period or sample size selection protocol; and studies that investigated complete pulpotomy treatment only, partial pulpotomy in primary teeth, or treatment of sound permanent posterior teeth.

Data Extraction

A flowchart of the search process and excluded/included articles was developed. Following the initial screening of all identified publications based on the title and abstract, the same five reviewers evaluated the full text of each article to be included. If the full text was not available, the relevant reviewer e-mailed the authors to obtain access. For each included study, the following information was entered into a Microsoft Excel spreadsheet: the final diagnosis, level of pulp tissue removal, pulp capping agent, follow-up interval/s, hemostatic solution, hemostasis duration, root apex status, success rate, attrition rate, type of final restoration, patient age, sample size, gender, study design, and main outcomes.

Quality and Risk Assessment of the Included Studies

The risk of bias in the included studies was assessed to appraise the strengths and limitations, and establish transparency regarding the evidence synthesis, analysis, and results. The quality of the included studies was assessed according to the specific study design, namely, randomized clinical trials. The risk of bias visualization (robvis) tool from the Cochrane Collaboration was used [25]. Risk evaluation was carried out separately by five reviewers. In the event of a dispute, a sixth reviewer was consulted. The quality of each domain was assessed as “low,” “unclear,” or “high.” These assessments were reported for each selected study. The overall risk of bias associated with each study was evaluated as follows: low risk of bias, all domains were assessed as “low risk”; unclear risk of bias, at least one domain was assessed as “unclear risk”; and high risk of bias, at least one domain was assessed as “high risk.”

Statistical Analysis and Synthesis of Results

Due to the heterogeneity of the included studies, a meta-analysis was not performed with this systematic review. The synthesized results were entered into one table. Different data items including preliminarily pulpal diagnosis, permanent restoration, pulp capping agent, age, apical closure, and study type were collated. All results were presented as descriptive data only.

Results

Study Selection and Characteristics

**Figure 1:**
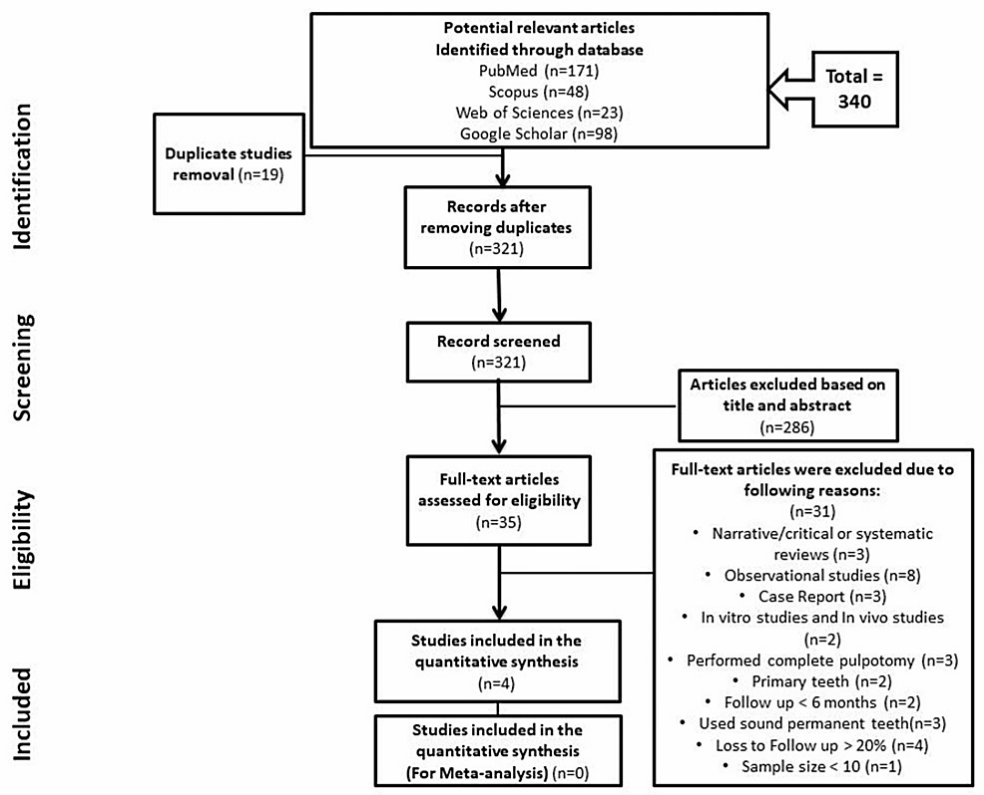
Flowchart of the PRISMA guidelines.

The study flowchart is shown in Figure 1 to explain the selection process. Four studies were included in this systematic review. Table 1 summarizes the characteristics of the included papers. All the included RCTs were conducted in different countries, including Jordan [16], Thailand [20], Korea [22], and Turkey [23]. In total, 292 patients, aged between six and 52 years old, were included. Only one study included teeth with irreversible pulpitis [16]. The other three studies investigated teeth with normal pulpal status or reversible pulpitis [20,22,23]. Five reviewers examined all four selected papers individually. Discrepancies were managed through discussion with a sixth reviewer.

**Table 1:** Summary of the characteristics of the included studies. RCT: randomized clinical trial; CH: calcium hydroxide; MTA: mineral trioxide aggregate-like material; SD: standard deviation

Study	Country	Study design	Sample size	Age (years) (mean±SD)	Follow-up months (mean±SD)	Pulp capping agent	Restoration	Apex	Preoperative pulp diagnosis
Taha et al. (2017) [16]	Jordan	RCT	50	20-52 (30.3±9.6)	6-24	CH and MTA	Amalgam and composite	Closed	Irreversible
Chailertvanitkul et al. (2014) [20]	Thailand	RCT	80	7-10	3-24	CH and MTA	Amalgam	Open	Normal/reversible
Kang et al. (2017) [22]	Republic of Korea	RCT	82	(29.3±14.8)	1-12	MTA	Composite indirect restoration	Closed	Normal/reversible
Özgür et al. (2017) [23]	Turkey	RCT	80	6-13 (8.57±1.25)	6-24 (23±3.98)	CH and MTA	Composite	Open	Normal/reversible

Success Rate of Partial Pulpotomy Treatment at Different Time Periods

The individual success rate of the four selected studies at six months ranged from 74% to 100% [16,20,22,23]. The combined success rate was 94% (Table 2). At one year, the individual success rate ranged from 70% to 99% [16,20,22,23], and the combined success rate was 93% (Table 2). At two years, the individual success rate from three of the studies was between 65% and 97% [16,20,23], and the combined success rate was 90% (Table 2). Success rates after six and 12 months were available from all four RCTs, but the success rate at 24 months was only reported by three out of the four selected studies [16,20,23]. The success rates reported by Taha et al. [16] were the lowest (74%, 70%, and 65% for the six-month, one-year, and two-year follow-up, respectively) when compared with the success rates of the other three studies [20,22,23]. Furthermore, teeth diagnosed with irreversible pulpitis showed the lowest success rate at different follow-up intervals [16] compared to teeth diagnosed with reversible pulpitis [20,22,23] (Table 2).

**Table 2:** Summary of the success rate of partial pulpotomy at different follow-ups. *: the number of patients after excluding those who did not return for follow-up; M: months, Y: year/years; NA: not applicable

Time period	Study	Sample size*	Number of success cases	Percentage of the success
6 M	Taha et al. (2017) [16]	46	34	74%
	Chailertvanitkul et al. (2014) [20]	84	84	100%
	Kang et al. (2017) [22]	83	80	96%
	Özgür et al. (2017) [23]	79	77	97%
Total	All the studies	292	275	94%
1 Y	Taha et al. (2017) [16]	44	31	70%
	Chailertvanitkul et al. (2014) [20]	79	78	99%
	Kang et al. (2017) [22]	71	68	96%
	Özgür et al. (2017) [23]	79	77	97%
Total	All the studies	273	254	93%
2 Y	Taha et al. (2017) [16]	49	32	65%
	Chailertvanitkul et al. (2014) [20]	78	76	97%
	Kang et al. (2017) [22]	NA	NA	NA
	Özgür et al. (2017) [23]	76	74	97%
Total	All the studies	203	182	90%

Quality and Risk Assessment of the Included Studies

**Figure 2:**
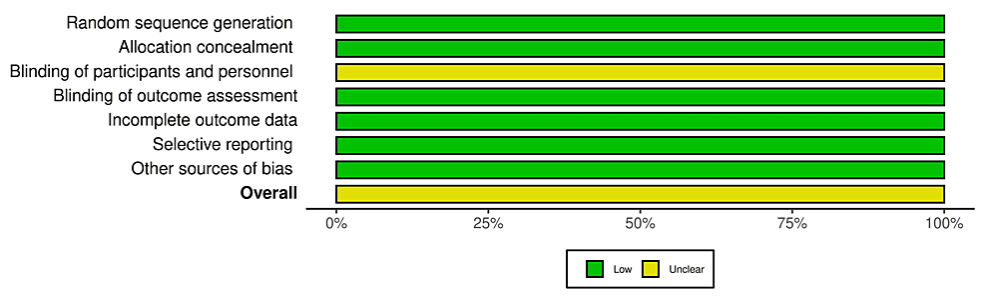
Risk of bias graph.

**Figure 3:**
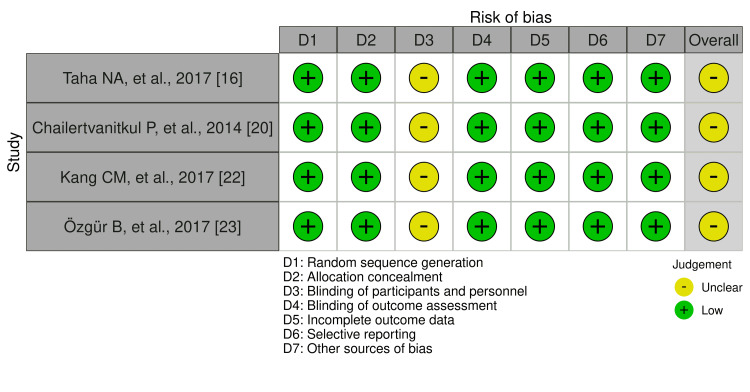
Risk of bias assessment using the robvis visualization tool.

The risk of bias was determined by five reviewers using a specific assessment tool (robvis) as mentioned earlier [25]. Most of the included studies had a low risk of bias in the following domains: random sequence generation (100%), allocation concealment (100%), blinding of outcomes assessment (100%), incomplete outcome data (100%), selective reporting (100%), and other sources of bias (100%). All four papers demonstrated an unclear risk of bias (100%) in the blinding of participants and personnel domain (Figure 2). Overall, all four included studies were judged to have an unclear risk of bias (100%) [16,20,22,23], as shown in Figure 3. The scoring of unclear risk of bias was given to all the papers because of the lack of sufficient information to make a clear judgment regarding the blinding of participants and personnel (Figure 3).

Discussion

Pulpotomy involves surgically removing the coronal pulp with the intention of preserving the vitality of the residual coronal or radicular pulpal tissues. A full pulpotomy or cervical pulpotomy has been shown to cause an inhibition of dentinal development, especially in immature permanent teeth, which can lead to root canal obliteration [26,27]. In contrast, partial pulpotomy aims to eliminate the injured and hyperemic dental tissues in immediate contact with the exposed pulp [28] and preserve the cellularly rich coronal pulpal tissues to provide a higher healing potential [10]. Treatment modalities such as partial pulpotomy have been recommended for asymptomatic teeth with carious pulpal exposures of 1-2 mm in diameter and bleeding that is controlled in less than two minutes [29].

Based on the findings of this systematic review, partial pulpotomy is an appropriate treatment choice for carious exposures in permanent posterior teeth. Based on available evidence, the prognostic outcome of partial pulpotomy relative to time remains unclear. Partial pulpotomy has a generally high success rate, independent of the different follow-up periods, as shown in Table 2. In a comparable procedure, Matsuo et al. [30] estimated 21 months as the appropriate timeframe for confirming a favorable prognosis using direct pulp capping. The same study determined three months to be an appropriate period for assessing preliminary prognosis, based on their observations that minimal changes occurred between three months and a year and a half. This is consistent with our results. The success rate did not differ significantly throughout the evaluation period (six, 12, and 24 months). Consequently, six months may be an appropriate interval for assessing the prognostic outcome of partial pulpotomy. Treatment could also be considered successful after a consensual period, Clinically, the American Dental Association recommends annual dental checkups for patients with low risk of oral diseases [31]. Radiographic evaluation is also important to verify treatment efficacy, and therefore, periodic radiographic examination is necessary.

According to Zanini et al. [32], the likely cause of failure or the microbial route should be indicated in longitudinal studies. Moreover, the restorative status and periodontal health at the time of treatment failure must be recorded. However, three of the papers considered in this systematic review [16,20,23] did not include this information. In the future, the periodontal health and restoration status of the treated tooth should be documented at the time of failure to assist with evaluating whether the failure was due to environmental causes or the vital pulp treatment itself. In our systematic review, final restorations were placed during the same visit, and periodontal health was documented in all four studies. Therefore, microbial contamination as a cause of failure is unlikely. As such, partial pulpotomy was considered the cause of failure.

The pulpal tissues near carious lesions are prone to localized permanent changes caused by bacteria. In contrast, the deeper residual pulp tissues are thought to be devoid of considerable inflammatory changes. Among the various variables that may affect treatment planning are the degree of infection and inflammation [31,32]. According to this systematic review, the only predictive factor of success is the preoperative pulp condition (Table 1). Moreover, the treatment decision regarding vital pulp therapy or root canal treatment is determined by the preliminary diagnosis. Furthermore, a diagnosis of irreversible pulpitis was associated with higher failure rates. The reviewers noted that only one paper [16] reported on the presumptive diagnosis of irreversible pulpitis, indicating that prudence is warranted when evaluating data on the efficacy of partial pulpotomy therapy for carious teeth. Since a sensitive and specific diagnostic method for determining the extent of inflammation is not available, the dental practitioner should continue to consider the bleeding cessation duration (less than two minutes) when deciding the preferred treatment option (partial pulpotomy, cervical pulpotomy, or endodontic treatment) [10].

A prolonged posttreatment observation duration increases the likelihood that a patient may be lost to follow-up. A loss to follow-up rate of over 20% jeopardizes the validity of the results [33]. Hence, we excluded studies with a follow-up rate of less than 80% from this systematic review. The investigation by Kang et al. [22] has been included despite an attrition rate of 26% at the one-year follow-up. We also attempted to select studies with a comparable clinical process. As a result, papers that applied various treatment strategies were eliminated. In a study of 15 teeth by Mejàre and Cvek [34], specific caries removal with CH was conducted before partial pulpotomy to avoid pulp exposures. The rationale was that before partial pulpotomy, a progressive strategy can affect the pulp-dentin complex defensive mechanism by generating secondary dentin production and inhibiting caries development [35,36].

Two of the selected RCTs [16,22] demonstrated that there were no significant differences in outcome between immature teeth with an open apex to mature teeth with a closed apex [20,23]. That is particularly important since vital pulp therapy has generally been suggested only for young patients with immature/open apices [12,37,38]. The findings of this systematic review suggest that partial pulpotomy may be applicable to both mature and immature permanent teeth.

Two of the four reviewed articles involved children [20,23], while the other two involved adults aged 29 years and above [16,22]. There have been ongoing debates [16,22] about the influence of age on the clinical success of vital pulp treatment. With improved procedures and a growing body of data supporting the use of bioengineered [39] or bioactive [40,41] materials for pulp therapy, the success of partial pulpotomy may also be improved, regardless of age.

This review has some limitations. This systematic review included only four randomized controlled trials. The type of final restoration (amalgam, composite, and stainless steel crown) differed between the studies. Regrettably, there was insufficient information to investigate the final restoration as a potential prognostic factor. In this review, the only identified prognostic factor was pulpal diagnosis. All four papers showed an unclear risk of bias. Furthermore, due to the heterogeneity of the included papers, a meta-analysis could not be performed.

The findings of this systematic review confirmed that partial pulpotomy is a viable treatment option for cariously exposed permanent posterior teeth. Partial pulpotomy showed a high success rate of 90% after 24 months. Preoperative pulp diagnosis is an important prognostic factor. Furthermore, irreversible pulpitis is associated with the lowest success rate.

## Conclusions

This systematic review concludes that the success rate of partial pulpotomy for the treatment of cariously exposed permanent posterior teeth is 90% after two years. The ability to precisely predict treatment success is hindered by the failure to determine the extent of pulpal inflammation and the possibility of the pulp healing following exposures. Nevertheless, advances in techniques and materials may enable dental practitioners to consider partial pulpotomy for preserving tooth vitality in the future. This would minimize the costs of endodontic treatment. More human clinical and radiographic studies to further assess the effectiveness of partial pulpotomy are warranted.
